# A cost utility analysis alongside a cluster-randomised trial evaluating a minor ailment service compared to usual care in community pharmacy

**DOI:** 10.1186/s12913-021-07188-4

**Published:** 2021-11-20

**Authors:** Noelia Amador-Fernández, Shalom I. Benrimoj, Leticia García-Mochón, Victoria García-Cárdenas, Sarah Dineen-Griffin, Miguel Ángel Gastelurrutia, Jesús Carlos Gómez-Martínez, Vicente Colomer-Molina, Fernando Martínez-Martínez

**Affiliations:** 1 Pharmaceutical Care Research Group, University of Granada, Campus de Cartuja, Granada, 18071 Spain; 2 Andalusian School of Public Health, Cuesta del Observatorio, Granada, 18080 Spain; 3 Graduate School of Health, University of Technology Sydney, Chippendale, NSW 2008 Australia; 4 Health Services Management, School of Biomedical Sciences, Charles Sturt University, Bathurst, NSW 2795 Australia; 5 Community pharmacist, Barcelona, Valencia, Spain

**Keywords:** Community pharmacy services, Primary health care, Self care, Self medication, Nonprescription drugs, Cost-utility analysis; minor ailment service

## Abstract

**Background:**

Minor ailments are “self-limiting conditions which may be diagnosed and managed without a medical intervention”. A cluster randomised controlled trial (cRCT) was designed to evaluate the clinical, humanistic and economic outcomes of a Minor Ailment Service (MAS) in community pharmacy (CP) compared with usual care (UC).

**Methods:**

The cRCT was conducted for 6 months from December 2017. The pharmacist-patient intervention consisted of a standardised face-to-face consultation on a web-based program using co-developed protocols, pharmacists’ training, practice change facilitators and patients’ educational material. Patients requesting a non-prescription medication (direct product request) or presenting minor ailments received MAS or UC and were followed-up by telephone 10-days after the consultation.

The primary economic outcomes were incremental cost-utility ratio (ICUR) of the service and health related quality of life (HRQoL). Total costs included health system, CPs and patient direct costs: health professionals’ consultation time, medication costs, pharmacists’ training costs, investment of the pharmacy and consultation costs within the 10 days following the initial consultation. The HRQoL was obtained using the EuroQoL 5D-5L at the time of the consultation and at 10-days follow up. A sensitivity analysis was carried out using bootstrapping. There were two sub-group analyses undertaken, for symptom presentation and direct product requests, to evaluate possible differences.

**Results:**

A total of 808 patients (323 MAS and 485 UC) were recruited in 27 CPs with 42 pharmacists (20 MAS and 22 UC). 64.7% (*n* = 523) of patients responded to follow-up after their consultation in CP. MAS patients gained an additional 0.0003 QALYs (*p* = 0.053). When considering only MAS patients presenting with symptoms, the ICUR was 24,733€/QALY with a 47.4% probability of cost-effectiveness (willingness to pay of 25,000€/QALY). Although when considering patients presenting for a direct product request, MAS was the dominant strategy with a 93.69% probability of cost-effectiveness.

**Conclusions:**

Expanding community pharmacists’ scope through MAS may benefit health systems. To be fully cost effective, MAS should not only include consultations arising from symptom presentation but also include an oversight of self-selected products by patients. MAS increase patient safety through the appropriate use of non-prescription medication and through the direct referral of patients to GP.

**Trial registration:**

ISRCTN, ISRCTN17235323. Registered 07/05/2021 - Retrospectively registered

**Supplementary Information:**

The online version contains supplementary material available at 10.1186/s12913-021-07188-4.

## Background

The World Health Organization (WHO) defines the pharmacy practice mission as “contributing to health improvement and helping patients with health problems to make the best use of their medicines” [1]. One of the six major components of this mission is identifying, managing and triaging health-related problems [1]. Minor ailments are defined as “common or self-limiting conditions which may be diagnosed and managed without a medical (i.e. doctor) intervention” [2]. Traditionally, patients present in community pharmacy (CP) for these conditions or alternatively self-select a non-prescription medication [3]. There are many countries where non-prescription medicines to symptomatically treat these minor ailments are exclusively available to the public through CPs [1, 4].

Minor ailment services (MASs) have been implemented in CPs in a number of countries as part of governments’ health policies [5–10]. The objective is to promote CPs as a first point of call for minor ailments, encouraging patients to seek care at the appropriate level within the health system [11] thus diminishing cost and allowing optimal use of health resources. A survey carried out in ten different countries in 2013 [12] stated that 63% of the population in southern European countries consulted a general practitioner (GP) when suffering a minor ailment. Studies have estimated that the percentage of GP visits due to minor ailments vary between 18 to 31.2% [13, 14], with 13.2% potentially manageable by CPs [15]. Furthermore, the Australian Institute of Health and Welfare (AIHW) calculated that 7.5% of the emergency department (ED) visits in Australia in 2018–2019 were due to non-urgent visits (triage category 5) [16]. A study carried out in North East England estimated that up to 8% [15] of ED workload is for minor ailment presentations that could potentially be managed in CP.

The economic impact of MAS has been previously compared to GP and ED settings [15, 17]. Across England and Scotland, studies have concluded that MASs release National Health Services (NHS) resources by redirecting care for minor ailments to lower costs settings such as CP [18, 19] with a total cost annual saving estimated to be approximately £1.1 billion (2013) [15]. In Canada, a pharmacist prescribing for minor ailment programs (PPMA) that addressed nine common minor ailments in the province of Ontario was estimated to save the health system more than $12.3 million [20]. The Conference Board of Canada calculated a healthcare cost reduction, by using CP for treating minor ailments and administering vaccines, between $100 million and $200 million from avoided GP and ED visits [21]. In an economic impact analysis in the province of Saskatchewan, PPMA resulted in an estimated health system cost saving of $546,832 [17].

However, the cost utility or cost effectiveness of MAS compared to usual care (UC) has been addressed in only one paper conducted in Australia [22]. The researchers showed that MAS demonstrated cost effectiveness compared to UC with an incremental cost-utility ratio (ICUR) of $2277 (95% CI, $681.49–3811.22) (Australian dollars) per QALY.

The application of MAS to the Spanish health system requires local data to ensure transferability. Since no cost utility evaluations have been carried out comparing MAS to UC in Spain, a study was designed to evaluate the clinical, humanistic and economic outcomes (ECHO [23]) of MAS compared with UC in CP. Humanistic and economic outcomes are reported in this paper.

## Methods

### Study design

The economic evaluation consisting of a cost utility analysis (CUA) was undertaken alongside a cluster randomised controlled trial (cRCT). The cRCT was conducted in Valencia (Spain) for 6 months between December 2017 and May 2018. The full report of the study in Spanish language is available [24].

### Participants

The Pharmaceutical Association of Valencia provided CPs with study information via phone or email. CPs within twenty-one municipalities agreed to participate. The municipalities were the clusters of the study to avoid contamination between groups. Municipalities with pharmacies who accepted to participate were randomised through a sequence of computer-generated random numbers to the control (UC) and the MAS arms applying a ratio of 1:1. Due to the nature of the intervention, pharmacists could not be blinded.

Patients aged ≥16 years or between 2 and 15 years of age if they were accompanied by a responsible adult, who were seeking care i.e. presenting symptoms or requesting a product (direct product request) for the minor ailments were included in the study. The minor ailments considered in the study were: dermatological problems (cold sore, foot fungi), gastrointestinal disturbance (diarrhoea, flatulence, heartburn or vomiting), pain (dysmenorrhea, headache, sore throat) and upper respiratory tract (cough, cold or nasal congestion).

### Description of the intervention (MAS)

The intervention is described using the TIDieR [25] checklist (Additional file 1). The main components to the intervention being:

1. A standardised pharmacist–patient consultation [26] using: co-developed with doctors’ management protocols for each specific symptom [27], patient educational material, and a web-based data collection software [28] that guided pharmacists through the consultation with selected pop-ups such as referral criteria (i.e. “red flag symptoms”).
2. Practice change facilitation (PCF): PCFs made regular on-site visits to MAS CPs to identify and resolve barriers with service provision and check the fidelity of the intervention.
3. Educational training for MAS pharmacists was a twelve-hour training session delivered prior to the beginning of the trial. It covered service provision, good practice standards, service protocols, communication’s skills with the patient and other health professionals, web-based data collection software use, data collection and trial protocol.

Patients attending UC pharmacies received usual pharmacist practice. Normally, in Spain, usual pharmacist practice does not include the use protocols or an IT system. When a patient presents in CP with a minor ailment or requesting a product, a consultation is carried. However, the depth and breadth of this consultation does vary. Also, pharmacists in the UC group did not receive the support of a practice change facilitator. Pharmacists in the control group attended a three-hour training on data collection procedures and patient recruitment.

### Study outcomes

Incremental cost-utility ratio (ICUR) of the service and health related quality of life (HRQoL) were the main economic and humanistic outcomes. A health system and patient perspective was chosen.

Data collection was undertaken at the moment of the consultation in the CP (i.e. patient demographics, medicines supplied, HRQoL) using a web-based data collection software [28]. Ten days after the consultation, patients were contacted by telephone by the research group to collect post consultation data (i.e. HRQOL, symptom resolution using a Likert scale from 1 “not at all” to 5 “completely”, reconsultation rates whenever the patient had to consult again for the same ailment during the 10 days after prior consultation in CP, type of reconsultation which could be CP, GP or emergency department). This follow-up time frame was considered appropriate given minor ailments are self-limiting conditions that should resolve within a short period of time. Patients self-reported HRQoL using EuroQoL 5D-5L self-complete version on paper at the time of the pharmacy consultation and 10 days later through telephone interview with a researcher using the EuroQoL 5D-5L telephone version [29].

In addition, the control group documented the consultation, which is not normally part of UC.

### Sample size

Sample size calculation was based on the clinical primary study outcomes. A 10% absolute increase in appropriate medical referral rate (85 to 95%) [30] and modification of direct product request (8 to 18%) [31, 32] were estimated. The sample size was calculated with ≥0.9 power, type I error rate of 5%, equal allocation ratio and assuming an intra-cluster correlation of 0.01. The larger of the two-estimated sample size calculations was used to determine the overall sample size, of 726 patients (allowing for 10% dropout).

### Statistical analysis and economic evaluation description

A descriptive analysis was performed to analyse baseline characteristics by group. Continuous variables were described using mean and standard deviation (SD) and categorical variables were presented as counts and percentages. Comparison of continuous variables between groups was undertaken using t-Student test and Kruskal-Wallis or Mann-Whitney (when skewed). Comparison of categorical variables was undertaken using Pearson’s χ2 tests. The level of statistical significance was established as *p* < 0.05.

The research was reported in accordance with the Consolidated Health Economic Evaluation Reporting Standards (CHEERS) checklist (Additional file 2) [33].

The effectiveness of the intervention was estimated as quality-adjusted life years (QALYs). QALYs were obtained from the cRCT [24] with the HRQoL questionnaire administered at the time of the pharmacy consultation using the EuroQoL 5D-5L self-complete version on paper and at 10-day follow-up via the telephone interview version by the research team [29]. Utility indexes associated with each health state were taken from the published Spanish tariff [34]. QALYs were calculated as area under the curve [35] considering a 10-day time horizon for patient follow-up. This method was implemented adding areas under the curve from geometrical forms obtained by lineal interpolation between utility values during the study period (10 days). Due to missing data in the sample in relation to the EQ-5D-5L data at follow-up, multiple imputation with chained equations was performed in order to allocate data. The multiple imputation missing data model included as predictive variables, EQ-5D-5L indices, at baseline and follow-up, and patient characteristics such as sex, age, and type of minor ailment (dermatological problems, gastrointestinal disturbance, pain and upper respiratory tract).

Firstly, the missing data were imputed under the missing at random (MAR) assumption; this was the main analysis (base-case scenario). Secondly, a completed case analysis was performed, assuming that patients who completed all follow-up were representative of the entire sample who initially agreed to participate. In this case, all participants who did not return the HRQoL questionnaire were removed, which assumes data is missing completely at random (MCAR). A simple pattern mixture model was implemented, following the approach recommended by Faria et al. [36] The utilities shown in both cases have been adjusted for age, gender, type of minor ailment, symptom duration and baseline utility.

Total direct costs included costs to the health system, CPs and direct patient costs. Direct costs included the cost of health professionals’ consultation time, medication costs, pharmacists’ training costs and investment of the pharmacy (infrastructure, etc.) and costs of patients’ re-consultation in the following 10 days after initial consultation in CP (including contacts with all health providers such as GP and ED consultations). To value each resource item in terms of its unit cost, additional data was used:

- Medication costs, as out-of-pocket costs borne by the patient, were based on official prices listed in the Bot Plus® database at September 2018 [37].
- CP consultation costs (for both groups) were calculated on time spent by pharmacists for the provision of the service, obtained from the pharmacists’ web-based data collection software for the cRCT. Considering pharmacist role (supervisor/regular pharmacist): time used for pharmacist-patient consultation was 8.00 min (SD = 2.45) for a supervisor in MAS group, 5.35 min (SD = 3.20) for a regular pharmacist in MAS group, 6.57 min (SD = 3.90) for a supervisor in UC group and 4.95 min (SD = 3.85) for a regular pharmacist in UC group. Pharmacists in UC group were asked to document the consultation which is not part of usual care.
- The unit cost of labour of the community pharmacist was calculated using the pharmacist’ salary as determined by the Spanish community pharmacy agreement [38]. Supervisor pharmacists’ salary was 0.315 €/minute and regular pharmacists’ salary was 0.293 €/minute.
- To calculate the costs related to the infrastructure investment and maintenance costs for the pharmacy, estimates were used from a previous Spanish study [39].
- Pharmacist training costs were for the twelve-hour course (classroom costs, presenter’s costs, travels and accommodation costs).
- GP (56.95€) and ED (105.27€) consultation costs were based on Valencian Law 20/2017 [40].In order to allocate the costs proportional to each patient, the mean cost of investment per pharmacy was divided by the mean estimated number of patients included in a potential MAS per CP. A study conducted by the General Pharmaceutical Council of Spain stated that 15–20% of patients attending community pharmacy presented with minor ailment symptoms or were self-selecting a medication for treatment of their symptoms [41]. Five thousand four hundred patients were estimated to potentially access a CP MAS annually with an estimated of approximately 120 patients per day per CP [42].

The investment of pharmacies in the UC group was assumed to be null.

Seemingly unrelated regression (SUR) was used to jointly model costs and QALYs.Cost and QALY were adjusted for sex, age, type of minor ailment, and QALYs were also adjusted for baseline utility to account for differences between groups at baseline. Coefficients were combined across the multiple imputed datasets using Rubin’s rules [36].

To assess the robustness of the results, a subgroup analysis and probability analysis was carried out alongside the missing data model described above. The two subgroups analysis accounting for presentation type were performed to evaluate possible differences. This included patients presenting to the CP with symptoms or patients directly requesting a product.

The probabilistic analysis assesses the uncertainty in cost and outcomes across both alternatives of the study (MAS and UC) and was conducted by bootstrapping cost and QALY pairs from each patient with 1000 replicates. The results of the replicates are presented in the cost-utility plane. The probability of the intervention being cost-effective was calculated assuming data was bivariate and normally distributed [43].

In addition, a cost-effectiveness acceptability curve (CEAC) was derived to estimate the probability of the intervention being cost-effective at different amounts of society’s willingness to pay for health outcomes. Since there is not a formal QALY monetary value assigned by the Valencian or National Spanish government, a study carried out by the government in Canary Island (Spain) [44] was used for the assignment of a monetary value to the QALY. They suggested a willingness to pay per QALY from €20,000 to €25,000. All analyses were performed using STATA 14 [45].

### Ethics approval and consent to participate

The study was approved by the University of Granada Ethics Committee (approval number 331/CEIH/2017) and Xátiva-Ontinyent Ethics Committee “Lluís Alcanyís”. All methods were carried out in accordance with relevant guidelines and regulations. Pharmacists were informed of the study and provided written consent to participate. Informed consent was obtained from all subjects and/or their legal guardian(s).

### Trial registration

ISRCTN, ISRCTN17235323. Registered 07/05/2021 - Retrospectively registered, https://www.isrctn.com/ISRCTN17235323

## Results

A total of 808 patients (13 MAS and 14 UC) were recruited in 27 CPs with 42 pharmacists (20 MAS and 22 UC) (characteristics are included in Additional file 3). Most patients were female (63.1%, *n* = 510), presented with upper respiratory tract symptoms (65.5%, *n* = 529), had a symptom presentation (69.8%, *n* = 564), presented symptoms suffered previously (91.6%, *n* = 740) and had not treated their symptoms for this episode prior to visiting the pharmacy (78.8%, *n* = 637). The mean patient age was 47.6 years old (SD = 16.6). Statistical differences between groups were only found for baseline HRQoL (both utility and EQ-VAS); the MAS group presented a utility index of 0.87 (SD = 0.12) compared to the UC group, of 0.89 (SD = 0.14) (*p* < 0.001). Patients visiting MAS pharmacies with direct product requests had lower baseline HRQoL (0.86, SD = 0.11) compared with UC patients (0.90, SD = 0.12) (*p* = 0.020).

Health system, CPs and patient direct costs are presented in Table 1. Thirty pharmacists attended each training sessions and thus the cost per pharmacist was 272.20€ (overall training cost was 3090€/30 pharmacists), with pharmacist’s time costing 169.20€. Approximately 5400 patients were estimated to potentially access a CP MAS annually per pharmacy [39] with a training cost per consultation of 0.05€. Training costs were only included for the MAS pharmacies.

**Table 1:** Summary of identified health resources and cost estimates

	MAS CP^a^ (***n*** = 323) Mean (SD^b^) (€)	UC CP^a^ (***n*** = 485) Mean (SD^b^) (€)	***P***-value
Medication cost^c^	7.67 (2.62)	7.88 (3.11)	0.331
Consultation cost^d^	1.55 (0.95)	1.13 (1,05)	0.000
Training cost	0.05 (0.00)	0.00 (0.00)	NA
Infrastructure and maintenance cost	0.53 (0.00)	0.53 (0.00)	NA
Total cost consultation in CP	9.80 (3.37)	9.54 (4.07)	0.012
Cost of other visits to GP or ED	11.61 (13.19)	4.94 (9.35)	0.001
Total cost^e^	21.41 (30.39)	14.48 (23.38)	0.001

^a^*UC CP* Usual Care Community Pharmacy, *MAS CP* Minor Ailment Service Community Pharmacy

^b^SD: Standard Deviation

^c^In Spain, medication costs are paid by the patient

^d^Considering pharmacist role (supervisor/regular pharmacist): time used for pharmacist-patient consultation was 8.00 min (SD = 2.45) for a supervisor in MAS group, 5.35 min (SD = 3.20) for a regular pharmacist in MAS group, 6.57 min (SD = 3.90) for a supervisor in UC group and 4.95 min (SD = 3.85) for a regular pharmacist in UC group. Pharmacists in UC group were asked to document the consultation which is not part of usual care

eFirst consultation in CP and other consultations during the 10 days studied were considered

*NA* Not applicable

The total infrastructure investment and maintenance costs were estimated to be €2883.43 per year with a cost per consultation of 0.53€.

**Fig. 1:**
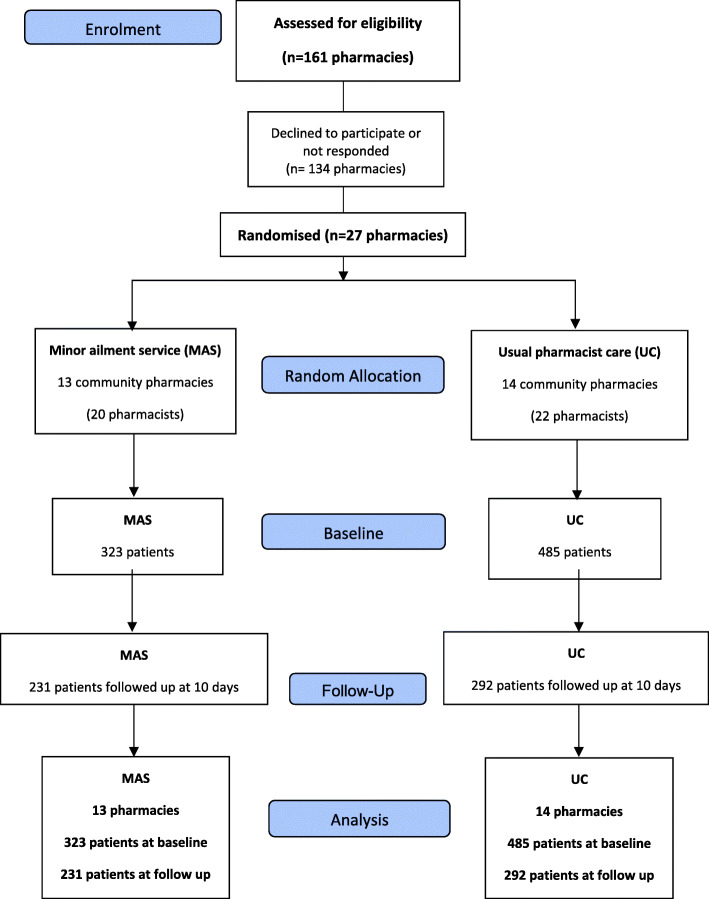
CONSORT 2010 Flowchart: Minor Ailment Service and usual pharmacist care

A total of 523 patients were followed up after consultation in CP (64.7% out of the 808 patients), 292 in UC pharmacies and 231 in MAS group (Fig. 1). The number of patients lost to follow-up was a total of 35.3% (*n* = 285). Within the MAS group this lost was 28.5% (92/323) compared to the UC group of 39.8% (193/485).

HRQoL was higher for those patients visiting MAS CPs with no statistically significant differences found between groups at follow-up (*p* = 0.053 without multiple imputation and *p* = 0.042 with multiple imputation) (Table 2).

**Table 2:** EuroQoL 5D-5L scores (adjusted)

ALTERNATIVES	GROUP	Number with missing baseline data	EQ-5D-5L baseline mean (SD^b^)^c^	Number with missing data at 10 days	EQ-5D-5L at 10 days mean (SD^b^)^c^
Without multiple imputation (complete case analysis)	UC CP^a^ (*n* = 285)	0	0.911 (0,010)	200	0.921 (0.011)
	MAS CP^a^ (*n* = 224)	2	0.891 (0,011)	101	0.922 (0.013)
	*P* value		0.170		0.053
With multiple imputation (base case)	UC CP^a^ (*n* = 285)	0	0.900 (0.008)	0	0.917 (0.018)
	MAS CP^a^ (*n* = 222)	0	0.881 (0.009)	0	0.926 (0.014)
	*P* value	0	0.051	0	0.042

^a^*UC CP* Usual Care Community Pharmacy, *MAS CP* Minor Ailment Service Community Pharmacy

^b^SD: Standard Deviation

^c^Adjusted by patient’s age and gender, minor ailment and symptom duration and baseline utility

When considering multiple imputation (base case analysis), the incremental cost-utility (ICUR) of MAS was 19,325€ per QALY with a 66.45% probability of cost-utility using a willingness to pay of 25,000€/QALY (Table 3). The results in the complete case scenario (without multiple imputation) had a slightly higher ICUR of 24,733€ per QALY.

**Table 3:** Cost utility analysis MAS CP vs. UC CP. Effectiveness measured as QALY

	Total Cost^b^	Cost Diff (mean)	QALY^b^	QALY Diff	ICER (€/QALY)	Probability of cost effectiveness		
						WTP 20,000€	WTP 25,000€	WTP 30.000
*All sample*								
*Multiple imputation (base case)*								
UC CP^a^	10.68		0.0246					
MAS CP^a^	17.19	6.51	0.0247	0.0003	19,325	52.39%	66.45%	75.36%
*Complete Cases*								
UC CP^a^	12.61		0.0245					
MAS CP^a^	20.03	7.42	0.0248	0.0003	24,733	36.19%	47.40%	60.10%
Subgroup with symptom presentation								
*Multiple imputation (base case for subgroup analysis)*								
UC CP^a^	10.35		0.0245					
MAS CP*	19.48	9.13	0.0247	0.0002	42,322	14.09%	23.92%	33.15%
*Complete Cases*								
UC CP^a^	12.59		0.0245					
MAS CP^a^	22.97	10.45	0.0248	0.0002	48,126	11.20%	17.39%	24.40%
Subgroup with direct product request								
*Multiple imputation (base case for subgroup analysis)*								
UC CP^a^	11.59		0.0242					
MAS CP^a^	11.90	0.31	0.0248	0.0006	523	96.83%	97.32%	97.58%
*Complete Cases*								
UC CP*	14.25		0.0247					
MAS CP^a^	13.54	−0.71	0.0249	0.0006	Dominant	92.60%	93.69%	94.49%

^a^*UC CP* Usual Care Community Pharmacy, *MAS CP* Minor Ailment Service Community Pharmacy

^b^Adjusted by patient’s age and gender, minor ailment, symptom duration and QALY at baseline (first consultation in CP and other consultations such as GP or ED visits during the 10 days studied were considered)

Thirty percent (*n* = 244) of patients self-selected a product to treat their minor ailment (Additional file 3). When cost-utility was studied for these patients, MAS was the dominant strategy with a 97.32% probability of cost-utility with a willingness to pay of 25,000€/QALY (Table 3).

**Fig. 2:**
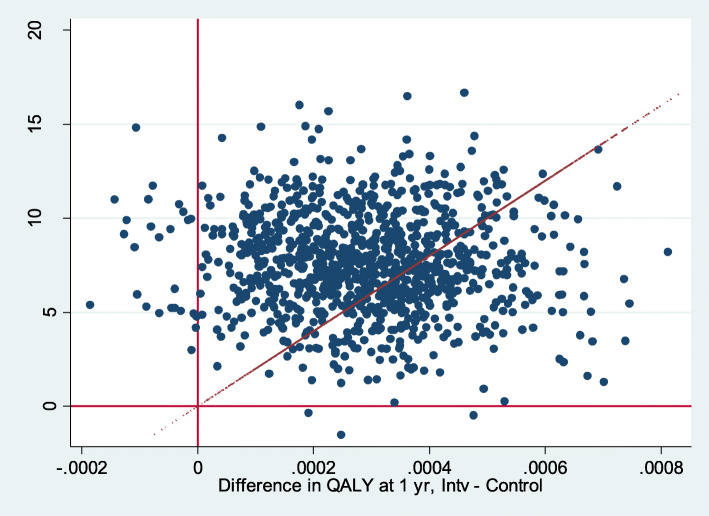
Bootstrap for both (symptom presentation and direct product request) and willingness to pay of 20,000€ (base case)

**Fig. 3:**
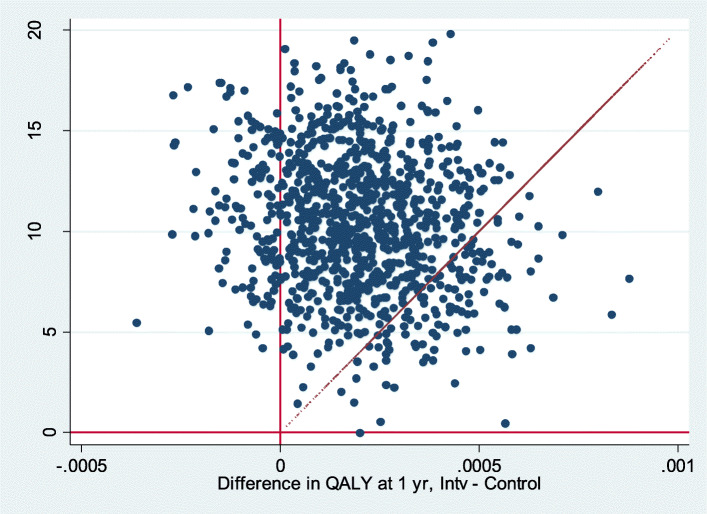
Bootstrap for symptom presentation and willingness to pay of 20,000€

**Fig. 4:**
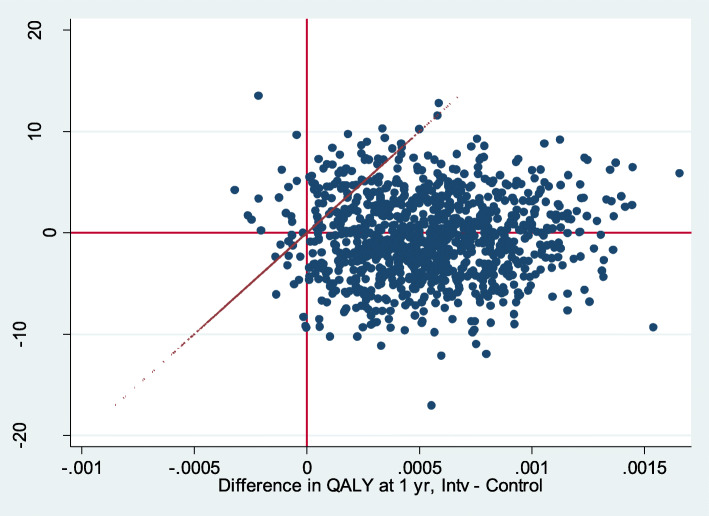
Bootstrap for direct product request and willingness to pay of 20,000€

Over one-third of the bootstrap simulations were located in the upper-right quadrant (53.29%) of the cost-utility plane with a willingness to pay of 20,000€/QALY (Fig. 2).Differences were found when only considering symptom presentation (Fig. 3) compared to direct product request presentation types (Fig. 4). When only considering direct product request, most of the bootstrap simulations were located under the threshold for a willingness to pay of 20,000€/QALY (96.83%) (Fig. 4).

**Fig. 5:**
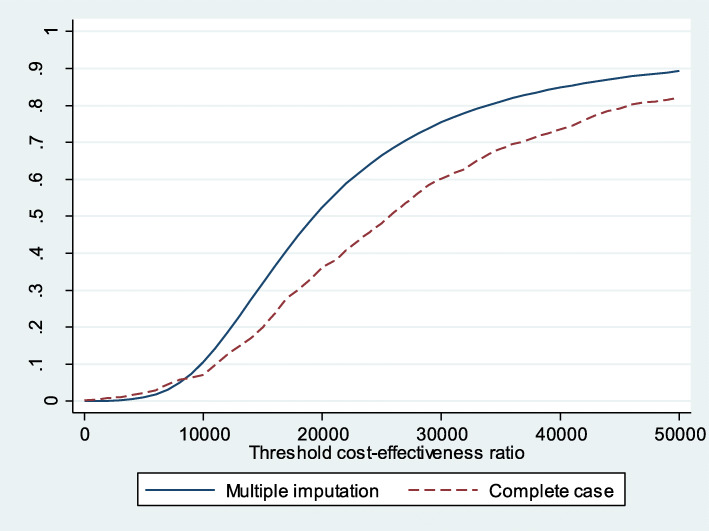
Acceptability curve MAS-UC (complete cases and multiple imputation)

**Fig. 6:**
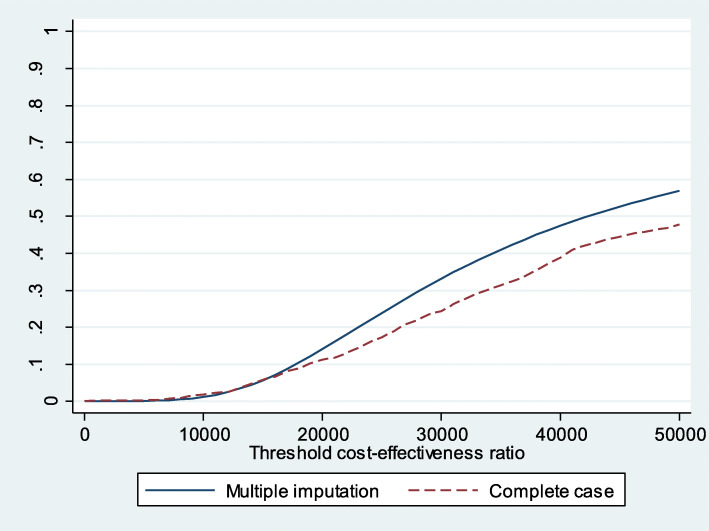
Acceptability curve MAS-UC (complete cases and multiple imputation for symptom presentation)

**Fig. 7:**
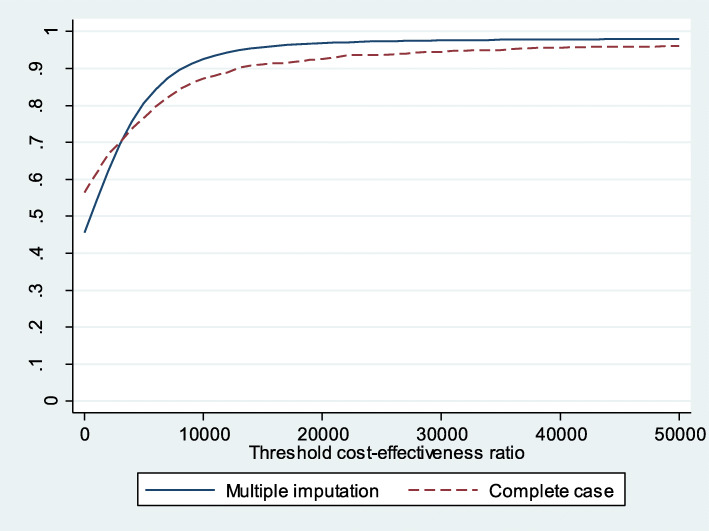
Acceptability curve MAS-UC (complete cases and multiple imputation for direct product request)

The acceptability curve shows that if the willingness to pay is 25,000€/QALY, the probability of MAS being cost effective, compared with UC, is 66.45% (Fig. 5). It decreases when considering symptom presentation (Fig. 6) and increases to 97.32% for consultations due to direct product requests (Fig. 7).

## Discussion

This is the first study to evaluate cost utility for a MAS in Spanish CPs compared to UC. The results of the study showed that implementing MAS has clinical benefits for patients, resulting in higher HRQoL as shown in previous studies [19, 22]. A positive effect was obtained in HRQoL when a ten-day time horizon was contemplated. Although, when MAS includes both, symptom presentation and direct product request, there is a low probability that MAS is cost-effective compared to UC. Minor ailments are self-limiting, they may have an impact in patients’ daily life so patients’ perception of their health status can change in a relative short period. In addition, MAS allows the provision of additional information about conditions or medicines, and referral to other health care professionals when needed.

When studying consultation costs, medication expensesaccounted for the main costs of the consultation. In Spain, the total costs of the medication bought without a prescription (over-the-counter medication) is paid by the patient. The cost of the medication was 80% of the total consultation cost.

Medication costs were similar in both groups. Pharmacists in the MAS group spent longer with the patient as would be expected due to the depth of the consultation. Total consultation costs (considering the first consultation in CP and any additional consultations within 10 days) was higher in the MAS group due to greater number of direct referrals to other health professionals. Some of the referral criteria used to refer patients in the agreed protocols were for example for “patients over 75 years old who had underling chronic conditions when suffering upper respiratory tract symptoms, long duration of symptoms (7 days without previous treatment or 72 h if treated without recovery) or high temperature (38 °C). Increasing appropriate referral may improve patient’s safety.

The results showed an ICUR of 19,325€/QALY due to the increase in QALY gain during 10 days after the consultation in MAS CPs compared to the UC group. These results are higher than obtained internationaly [22] (AUD $2277/ QALY) and may be due follow-up occurring at 10 days in this study, and at 14-days in the Australian study. Since the QALYs were estimated over a 10-day period, the gains for MAS CP group could have extended beyond the 10-day period.

Different studies [11, 15–17] have proven that when a MAS is implemented, it reduces GP consultations and decrease the number of patients presenting minor ailment symptoms at primary health care centres due to the shift of consultations between settings. Our study results showed that it is important to extend this service to direct product requests since the evidence suggests that there is increase in HRQoL, cost effectiveness and patient safety. MAS pharmacists modified by 11.4% [24] (compared to 4.5% in UC CP) the medication requested by the patients. Using the willingness to pay threshold of 25,000€, MAS was the dominant strategy with a 97.32% probability of cost-utility for patients with direct product requests.

As shown in previous studies, consultations were performed differently when symptoms were presented than for a direct product request [46]. When a patient presents with symptoms and is directly asking for advice, it provides an easier opportunity for the pharmacist to conduct a deeper consultation. The self-selection of the product by patient makes it challenging for pharmacists to intervene and in usual practice less advice is reportedly given to those patients [47, 48]. It appears that CPs are more engaged when resolving symptom presentations than when dealing with product requests [47, 48]. As this study showed, using protocols to deal with direct product requests has a greater effect on HRQoL, and therefore, MAS is more likely to be cost-effective than when only considering symptom presentations. Implementing quality standards in CP helps promote the safe and effective management of minor ailments [1, 49] and appropriate self-selection of non-prescription medications. MAS also limited the variability in community pharmacy consultations through training and the use of a standard process.

While this study only focussed on MAS versus UC, comparing MAS versus GP and ED visits in Spain would be valuable. The international literature suggests that implementing MAS may reduce the burden in GP and ED visits decreasing primary health spending [15, 17–19, 22]. In Spain, it has been suggested that CPs could help reduce primary care pressure through managing minor ailments [50]. In Spain in 2018, 230.6 million GP consultation and 28.7 million ED consultations took place [51]. Health expenditure in 2018 was 71,145 million € with only 14.6% (10,387 million €) intended to primary health care [52]. A Spanish national study carried out in 2018 concluded that 86.8% of the population visited the GP during the 12 months before and 15.4% over 15 years old could not get an appointment with their GP on time [53]. In Spain, GP consultations increased by 17% and by 18% for ED presentations between 1987 and 2017 [54]. Therefore, the implementation of MAS may lead to a more efficient use of the primary health care resources.

### Methodological limitations

There are a number of methodological limitations for this study:

The study took place in the province of Valencia, limiting the generalizability of results to a national level. The number of patients lost to follow-up was 35.3% (*n* = 285), it is interesting to note that a greater percentage of MAS patients responded to follow-up 10 days after the consultation, compared to the UC group. This may be due to the increased time spent with the patient during the MAS consultation.

Due to the impossibility of following patients up daily, the period established to contact patients after consulting the CP was 10 days, in some cases, the minor ailments did not fully resolve within the 10-days period (39.6%) and recovered before 10 days (60.4%). This could represent a limitation to our study as we took, for practical reasons, a 10-days period for the second measurement of the QALY.

Data obtained for consultations to GP and ED that took place during the 10 days after the initial consultation in CP were self-reported by the patient and not validated with official data. Future studies should contemplate the use of health system data to obtain a more reliable health system cost and should consider the inclusion of productivity costs and costs of any prescribed medications in subsequent consultations. PCFs costs were not included due to the limited time spent with community pharmacists and lack of reliable documentation.

The UC group was asked to document the consultation. Therefore, consultation time and consultation costs in the UC group may be overestimated.

## Conclusion

This is the first Spanish study to evaluate the economic impact of a MAS in CP, obtaining similar results to those carried out internationally. The overall findings showed that there is significant potential to adopt a MAS in Spain with an addition of including direct product request so that it has a higher impact on the health system. MAS may add further economic benefits due to the appropriateness of self-medication with non-prescription medicines. Expanding community pharmacists’ scope through MAS may benefit health systems; therefore, the contribution by CPs to this aspect of primary health care should not be underestimated.

## Supplementary Information

**Additional file 1.** TIDieR Checklist (Template for Intervention Description and Replication).

**Additional file 2.** CHEERS Checklist (Consolidated Health Economic Evaluation Reporting Standards).

**Additional file 3.** Baseline characteristics for the sample by pharmacy group.

## Data Availability

The datasets used and/or analysed during the current study are available from the corresponding author on reasonable request.

## Abbreviations

AIHW: Australian Institute of Health and Welfare
CHEERS: Consolidated Health Economic Evaluation Reporting Standards
CP: Community pharmacy
CRCT: Cluster randomised controlled trial
CUA: Cost utility analysis
ED: Emergency department
FIP: International Pharmaceutical Federation
GP: General medical practitioner
HRQoL: Health related quality of life
ICUR: Incremental cost-utility ratio
MAR: Missing at random
MAS: Minor ailment service
MCAR: Missing completely at random
NHS: National Health Services
PCF: Practice change facilitation
PPMA: Pharmacist prescribing for minor ailment programs
QALY: Quality-adjusted life years
SD: Standard deviation
UC: Usual pharmacist care
WHO: World Health Organization

## References

[1] The legal and regulatory framework for community pharmacies in the WHO European Region. Copenhagen: WHO Regional Office for Europe; 2019. Licence: CC BY-NC-SA 3.0 IGO.

[2] Jones R, White R, Armstrong D, Ashworth M, Peterset M (2010). Managing acute illnesses: an enquiry into the quality of general practice in England.

[3] Pharmaceutical Group of European Union (PGEU). The Community Pharmacy contribution to Sustainable Health Systems. [Revised sept 2020]. Available from: https://www.pgeu.eu/wp-content/uploads/2019/07/171123E-PGEU-Opinion-Paper-on-Sustainable-Health-Systems.pdf. Last access on oct 2021.

[4] Alves da Costa F, Foppe Van Mil JW, Alvarez-Risco A (2019). The pharmacist guide to implementing pharmaceutical care.

[5] Aly M, García-Cárdenas V, Williams K, Benrimoj SI (2018). A review of international pharmacy-based minor ailment services and proposed service design model. RSAP.

[6] Scottish Executive (2006). National Health Service (Scotland) ACT 1978 health board additional pharmaceutical services (minor ailment service) (Scotland) directions.

[7] Northern Ireland Executive (2010). Agreement reached on minor ailments service.

[8] Cym Taf University Local Health Board. New Choose Pharmacy Scheme. Cwm Taf Local Health Board. 2013. [Revised oct 2021]. Available from: http://www.cwmtafuhb.wales.nhs.uk/news/29092.

[9] Department of Health (2005). Implementing the new community pharmacy contractual framework.

[10] Canadian Pharmacists Association (2014). Summary of pharmacists’ expanded scope of practice across Canada.

[11] Bednall R, McRobbie D, Duncan J, Williams D (2003). Identification of patients attending accident and emergency who may be suitable for treatment by a pharmacist. Fam Pract.

[12] European Platform for Patients’ Organisations, Science and Industry (Epposi) (2013). The Epposi Barometer: Consumer Perceptions of Self Care in Europe.

[13] Proprietary Association of Great Britain (PAGB) (2008). PAGB annual review.

[14] Watson MC, Holland R, Ferguson J, Porteous T, Sach T, Cleland J (2014). Community pharmacy Management of Minor Illness (the MINA study).

[15] Fielding S, Porteous T, Ferguson J, Maskrey V, Blyth A, Paudyal V, Barton G, Holland R, Bond CM, Watson MC (2015). Estimating the burden of minor ailment consultations in general practices and emergency departments through retrospective review of routine data in north East Scotland. Fam Pract.

[16] Australian Institute of Health and Welfare (2020). Use of emergency departments for lower urgency care: 2015–16 to 2018–19. Cat. no. PHC 3.

[17] Rafferty E, Yaghoubi M, Taylor J, Farag M (2017). Costs and savings associated with a pharmacists prescribing for minor ailments program in Saskatchewan. BMC.

[18] Baqir W, Learoyd T, Sim A, Todd A (2011). Cost analysis of a community pharmacy ‘minor ailment scheme’ across three primary care trusts in the north east of England. J Public Health.

[19] Watson MC, Ferguson J, Barton GR, Maskrey V, Blyth A, Paudyal V, Bond CM, Holland R, Porteous T, Sach TH, Wright D, Fielding S (2014). A cohort study of influences, health outcomes and costs of patients’ health-seeking behaviour for minor ailments from primary and emergency care settings. BMJ Open.

[20] Chain Drug Review. Empower Ontario pharmacists to do more. [Revised sept 2020]. Available from: http://www.chaindrugreview.com/study-empower-ontario-pharmacists-to-do-more/

[21] 9000 Points of Care: Improving access to affordable healthcare. Broader pharmacy’s five creative initiatives to improve healthcare system outcomes, deliver greater value and improve the patient experience. [Revised sept 2020]. Available from: http://9000pointsofcare.ca/wp-content/uploads/The-Plan.pdf

[22] Dineen-Griffin S, Williams KA, Vargas C, Benrimoj SI, Garcia-Cardenas V (2020). Cost utility of a pharmacist-led minor ailment service compared with usual pharmacist care. Cost Eff Resour Alloc.

[23] Kozma CM, Reeder CE, Schulz RM (1993). Economic, clinical, and humanistic outcomes: a planning model for pharmacoeconomic research. Clin Ther.

[24] Amador-Fernández N, Baixauli-Fernández VJ, Climent-Catalá MT, Colomer-Molina V, García-Agudo O, García-Cárdenas, et al. INDICA+PRO, informe sobre la evaluación del impacto clínico, humanístico y económico del servicio de indicación farmacéutica en el ámbito de la farmacia comunitaria. [INDICA+PRO: report for the clinical, humanistic and economic evaluation of a minor ailment service in community pharmacy] Granada: Grupo de Investigación en Atención Farmacéutica; 2019.

[25] De Barra M, Scott C, Johnston M, De Bruin M, Scott N, Matheson C (2018). Do pharmacy intervention reports adequately describe their interventions? A template for intervention description and replication analysis of reports included in a systematic review. BMJ Open.

[26] Pharmaceutical Care Forum in Community Pharmacy (2019). Practical guide to Pharmaceutical Care Services in Community Pharmacy.

[27] Amador-Fernández N, Amariles P, Baixauli-Fernández VJ, Benrimoj SI, Climent-Catalá MT, Colomer-Molina V (2018). Protocolos de Indicación Farmacéutica y Criterios de Derivación al Médico en Síntomas Menores [Protocols for the minor ailment service and referral criteria for minor ailments].

[28] Spanish Society of Community Pharmacy (SEFAC) (2016). SEFAC e_XPERT, software for the record of community pharmacy services.

[29] EuroQol Research Foundation. EQ-5D-5L. [Revised sept 2020]. Available from: https://euroqol.org/eq-5d-instruments/eq-5d-5l-about/

[30] Ocaña A. Efectividad del proceso estructurado de asesoramiento en síntomas menores frente al asesoramiento habitual en Farmacias Comunitarias españolas [effectiveness of a structured process for the management of minor ailments compared to usual care in community pharmacy] [doctoral thesis]. Granada: University of Granada; 2011.

[31] Paluch E, Jayawardena S, Wilson B, Farnsworth S (2016). Consumer self-selection, safety, and compliance with a novel over-the-counter ibuprofen 600-mg immediate-release and extended-release tablet. JAPhA.

[32] Eickhoff C, Hammerlein A, Griese N, Schulz M (2012). Nature and frequency of drug-related problems in self-medication (over-the-counter drugs) in daily community pharmacy practice in Germany. Pharmacoepidemiol Drug Saf.

[33] Husereau D, Drummond M, Petrou S, Carswell C, Moher D, Greenberg D, Augustovski F, Briggs AH, Mauskopf J, Loder E (2013). Consolidated health economic evaluation reporting standards (CHEERS)—explanation and elaboration: a report of the ISPOR health economic evaluations publication guidelines good reporting practices task force. Value Health.

[34] Ramos-Goñi JM, Pinto-Prades JL, Oppe M, Cabasés JM, Serrano-Aguilar P, Rivero-Arias O (2017). Valuation and modeling of EQ-5D-5L health states using a hybrid approach. Med Care.

[35] Drummond MF, O'brien BJ, Stoddart GL, Torrance GW (2001). Métodos Para la evaluación económica de los programas de asistencia sanitaria. [methods for the economic evaluation of health services].

[36] Faria R, Gomes M, Epstein D, White IR (2014). A guide to handling missing data in cost-effectiveness analysis conducted within randomised controlled trials. PharmacoEconomics.

[37] Consejo General de Colegios Oficiales de Farmacéuticos (CGCOF) (2018). Bot Plus. Base de Datos del Conocimiento Sanitario.

[38] Ministerio de Empleo y Seguridad Social. Resolución de 9 de abril de 2014, de la Dirección General de Empleo, por la que se registra y publica el laudo arbitral para oficinas de farmacia. Boletín Oficial del Estado núm. 112, de 8 de mayo de 2014, páginas 35242 a 35266. BOE-A-2014-4846.

[39] Consejo General de Colegios Oficiales de Farmacéuticos (CGCOF). Proyecto ConSIGUE para medida del impacto clínico, económico y humanístico del servicio de Seguimiento Fármaco-Terapéutico en mayores polimedicados, en la Farmacia Comunitaria española. [Consigue program for the clinical, economic and humanistic evaluation of a medication review with follow up in older patients in community pharmacy] Madrid: CGCOF; 2014.

[40] Valencian law 20/2017, 28th December. [2017/12159] (DOGV num. 8202 of 30.12.2017) Database reference 011728/2017. [Revised sept 2020]. Available from: https://www.boe.es/buscar/pdf/2018/BOE-A-2018-1870-consolidado.pdf

[41] Consejo General de Colegios Oficiales de Farmacéuticos (2002). Valoración del consejo sanitario en las oficinas de farmacia. [Evaluation of health education in community pharmacy].

[42] Vilanova O (2018). DMF: de 2010 a 2018, nueve años afianzando la relación entre el farmacéutico y la sociedad.

[43] O’Brien BJ, Briggs AH (2002). Analysis of uncertainty in health care cost-effectiveness studies: an introduction to statistical issues and methods. Stat Methods Med Res.

[44] Vallejo-Torres L, García-Lorenzo B, Castilla I, Valcárcel Nazco C, García-Pérez L, Linertová R, et al. Valor Monetario de un Año de Vida Ajustado por Calidad: Estimación empírica del coste de oportunidad en el Sistema Nacional de Salud. Canarias: Ministerio de Sanidad, Servicios Sociales e Igualdad; 2015.

[45] StataCorp LLC (2020). STATA 14.

[46] Eikenhorst L, Salema N, Anderson C (2017). A systematic review in select countries of the role of the pharmacist in consultations and sales of non-prescription medicines in community pharmacy. RSAP.

[47] Vella E, Azzopardi LM, Zarb-Adami M, Serracino-Inglott A (2009). Development of protocols for the provision of headache and back-pain treatments in Maltese community pharmacies. Int J Pharm Pract.

[48] Berger K, Eickhoff C, Schulz M (2005). Counselling quality in community pharmacies: implementation of the pseudo customer methodology in Germany. J Clin Pharm Ther.

[49] Inch J, Porteous T, Maskrey V, Blyth A, Burr J, Cleland J (2017). It's not what you do it's the way that it's measured: quality assessment of minor ailment management in community pharmacies. IJPP.

[50] Grupo de trabajo de instituciones farmacéuticas, sociedades científicas, distribución farmacéutica y expertos (2019). La farmacia en el sistema sanitario actual: Aportación a los retos demográficos y asistenciales [Community pharmacy in the health system nowadays: contribution to demographic and healthcare challenges].

[51] Ministerio de Sanidad, Consumo y Bienestar Social. Sanidad en datos, Año 2018. [Health with data, year 2018]. [Revised sept 2020]. Available from: https://www.mscbs.gob.es/estadEstudios/sanidadDatos/tablas/tabla17.htm

[52] Ministerio de Sanidad, Consumo y Bienestar Social. Estadística de gasto sanitario público 2018: Principales resultados. [Public health expenditure statistics 2018: Main results]. [Intenet] [Revised sept 2020]. Available from: https://www.mscbs.gob.es/estadEstudios/estadisticas/docs/EGSP2008/egspPrincipalesResultados.pdf

[53] Ministerio de Sanidad, Consumo y Bienestar Social. Encuesta Nacional de Salud, España 2017, Principales resultados. [National Health Survey, Spain 2017, Main results]. [Revised sept 2020]. Available from: https://www.mscbs.gob.es/estadEstudios/estadisticas/encuestaNacional/encuestaNac2017/ENSE2017_notatecnica.pdf

[54] Redacción médica. Encuesta Nacional de Salud 2017: máximo histórico de consultas y visitas a urgencias. [National Health Survey 2017: historical maximum of consultations and visits to the emergency departments]. [Revised sept 2020]. Available from: https://www.redaccionmedica.com/secciones/sanidad-hoy/encuesta-de-salud-2017-maximo-historico-de-consultas-y-visitas-a-urgencias-5304

