# Supplementary material for: A cost utility analysis alongside a cluster-randomised trial evaluating a minor ailment service compared to usual care in community pharmacy
Source: BMC Health Serv Res. 2021 Nov 20;21:1253. doi: 10.1186/s12913-021-07188-4 (PMC8605551; doi:10.1186/s12913-021-07188-4)
Supplement: Supplementary file 1 — Additional file 1. TIDieR Checklist (Template for Intervention Description and Replication). [file 12913_2021_7188_MOESM1_ESM.docx]

**
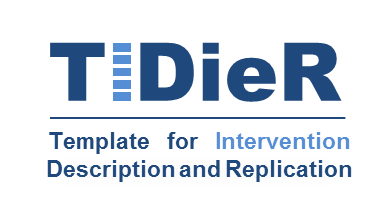
The TIDieR (Template for Intervention Description and Replication) Checklist*:**

Information to include when describing an intervention and the location of the information

| **Number** | **Item** | **Where located **** |
| --- | --- | --- |
| **1.** | **BRIEF NAME** | Minor ailment service (MAS). |
| **2.** | **WHY** | MAS is defined as a “professional service provided upon patient’s request in the pharmacy when unsure of which medicinal product to acquire […] for a specific health problem”^[[1]](#endnote-1)^. The international literature reports that structured minor ailments schemes improve the clinical, humanistic and economic outcomes for patients and the health care system^[[2]](#endnote-2),^^[[3]](#endnote-3),^^[[4]](#endnote-4)^. Collaboratively agreed protocols have been shown to enhance appropriate referrals by pharmacist to other health care professionals, particularly to general medical practitioner. Appropriate referrals increase the quality of the service and increase patient’s safety. Monitoring patient self-selection of non-prescription medication enhances quality use of these medicines and increases patient safety.  At present there appears to be wide variability of practices on how pharmacist respond to patients presenting symptoms and to self-medication product request. Quality standards need to be applied to promote the safe and effective management of minor ailments in community pharmacy setting. |
| **3.** | **WHAT** | The intervention was composed of:  1. Standardised consultation on an IT platform for pharmacist–patient intervention which included:   - 1. A MAS was provided following good pharmacy standards by the Pharmaceutical Care Forum in Community Pharmacy (see Procedures section).   2. Each minor ailment studied had a collaboratively agreed protocol (see Procedures section).   3. Educational material for the patient included non-pharmacological treatment for each minor ailment: [https://www.sefac.org/system/files/2020-01/INDICA%2BPRO_Informe.pdf](about:blank) (Appendix 4, pages 151 to 157)   4. An IT practice program led the pharmacists through the individual patient consultation with selected pop ups including protocol flow and referral criteria for each minor ailment. It guided pharmacists through two main pathways: patients presenting with symptoms or requesting a non-prescription medicine. An example can be found on: [https://www.sefac.org/system/files/2020-01/INDICA%2BPRO_Informe.pdf](about:blank) (Appendix 7, pages 160 to 163).  1. A practice change facilitator (PCF) made regular on-site visits during the study to resolve doubts, problems, advice, educate pharmacists and check fidelity of the intervention through data inspection. Support was also provided via email/telephone during the study period. PCF was trained to ensure the study objectives were met.   Educational training for pharmacists: three half-day course (12 hours) was delivered by two experts (a community pharmacist and a general medical practitioner) which included MAS procedure, good practice standards, agreed service protocols, communication’s skills with the patient and other health professionals and data collection methods. |
| **4.** | **Procedures** | Pharmaceutical Care Forum in Community Pharmacy in 2010 has standardized procedures for the delivery of a MAS in community pharmacy. These were adapted and enhanced in the IT consultation process  [https://www.portalfarma.com/inicio/serviciosprofesionales/forofarmaciacomunitaria/Documents/Practical%20Guide%20to%20Pharmaceutical%20Care%20Services%20-%20Forum%20Community%20Pharmacy%20pdf.pdf](about:blank) (Page 39 to 43)  https://www.portalfarma.com/Profesionales/Buenas-practicas-profesionales/Documents/BBPP-02-ENG-Servicio-Indicacion-Medicamentos.pdf  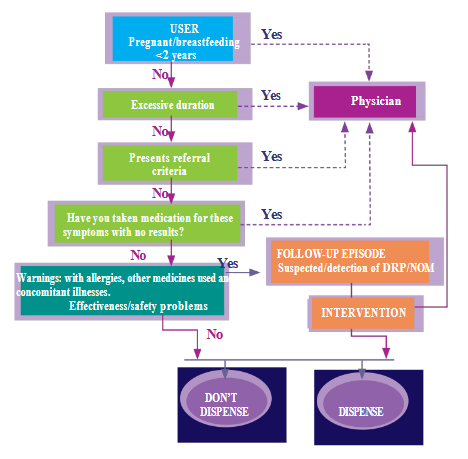  Figure 1. Procedure for Minor illness Service  The flow chart may be adapted to the specific circumstances of each patient and health problem  *DRP: Drug related problems / NOM: Negative outcomes related to medicines  Pharmaceutical Care Forum in Community Pharmacy developed this standard operational procedure (SOP) and established the requirements for providing MASs in the document “Good pharmacy practice in Spanish community pharmacy”.  Specific protocols for each symptom are published in “Protocols for the MAS and Referral Criteria for Minor Ailments”. These protocols and guideline were codesigned and agreed between community pharmacists and general medical practitioners and include referral criteria according to patient’s age, symptoms’ duration, red flags, other health problems and special physiological situations such as pregnancy, breastfeeding; appropriate pharmacological and non-pharmacological treatment for each specific minor ailment. An example for one of the minor ailment’s protocol (nasal congestion) can be found in Appendix 1 of: N. Amador-Fernández, S.I. Benrimoj, V.J. Baixauli, M.T. Climent, V. Colomer, O. Esteban, B. Fernández, O. García, V. García, J.I. García, M.A. Gastelurrutia, J.C. Gómez, F. Valls, F. Martínez, Colaboración farmacéutico-médico en la elaboración de protocolos consensuados para el tratamiento de síntomas menores: programa ‘INDICA+PRO’, Farmacéuticos Comunitarios. 11 (2019) 21-31. https://doi: 10.5672/FC.2173-9218.(2019/Vol11).004.03. |
| **5.** | **WHO PROVIDED** | Registered pharmacists provided the MAS after attending a 12-hour training and following the advice of a PCF.  Pharmacy staff other than the pharmacists were not included in the study. |
|  | **HOW** | MAS was provided through a face-to-face encounter between the pharmacist and the patient, so individual interviews were carried out in the community pharmacy. When patients attended the pharmacy either requesting a direct product request (non-prescription medicine) of presenting symptoms covered in the study they were informed about the study. If they accepted to participate the signed a consent form.  10 days following this consultation a researched phoned them at the number provided. |
| **6.** |  |  |
| **7.** | **WHERE** | Community pharmacies of twenty six municipalities in the region of Valencia (Spain): Agullent, Aielo de Malferit, Albaida, Alcudia de Crespins, Aldaia, Benaguasil, Benigánim, Bétera, Bocairent, Buñol, Canals, Chiva, El Palomar, Godelleta, L’Eliana, L’Ollería, Manises, Ontinyent, Puçol, Quart de Poblet, Riba-roja de Túria, Sagunt, Terrateig, Vilamarxant, Villanueva de Castellón, Xátiva. |
| **8.** | **WHEN and HOW MUCH** | The intervention was provided by the community pharmacist in a single consultation that took place when the patient visited the pharmacy asking for advice or requesting a non-prescription medication for one of the minor ailments included (see Tailoring).  The consultation using an IT practice program took a mean time of 8.00 minutes (SD=2.45) for superintendent pharmacists in the intervention group, 5.35 minutes (SD=3.20) for regular pharmacists in the intervention group, 6.57 minutes (SD=3.90) for superintendent pharmacists in the control group and 4.95 minutes (SD=3.85) for regular pharmacists in the control group. |
| **9.** | **TAILORING** | Patients were eligible if they were aged ≥ 16 years, and between two and 15 years of age accompanied by a responsible adult, able to provide informed consent and willing to be contacted by telephone 10 days after the consultation. Duration of the consultation was one of the study outcomes. Twelve different protocols were used, one for each minor ailment. The minor ailments studied were dermatological problems (cold sore, foot fungus), gastrointestinal disturbance (diarrhoea, flatulence, heartburn or vomiting), pain (dysmenorrhea, headache, sore throat) and upper respiratory tract-related conditions (cough, cold or nasal congestion) as they are some of the most frequent minor ailments consulted in community pharmacy. These guidelines included referral criteria and treatment to be evaluated depending on patient’s characteristics.  Standardised consultation could follow two main pathways depending on patients presenting with symptoms or requesting a medicine for a minor ailment. Those cases where the patient requested a medicine, the pharmacists had to evaluate if the medication requested was the most appropriate treatment. |
| **10. ^ǂ^** | **MODIFICATIONS** | No changes were made in the intervention during the course of the study. |
| **11.** | **HOW WELL (planned)** | The MAS scheme was codesigned with pharmacist, general medical practitioners, patients’ organisations and local health administrators using existing materials and nationally agreed documentation. The specific pharmacist/patient interventions were developed with this group over a six-month period and then piloted.  A PCF was trained to follow up the intervention and control pharmacists. He/she made monthly on-site visits in every community pharmacy to assess adherence to the guidelines. The PCF completed a checklist at each pharmacy including the collection of facilitators and barriers for delivering the service. The PCF check the fidelity of the intervention through checks on the IT program and the extracted data from this program. The PCF was available telephone and email contacts to assist pharmacists in the intervention group throughout the study. |
| **12.^ǂ^** | **HOW WELL (actual)** | In addition to further assess intervention adherence and fidelity, IT data collection program was checked by the research group. The consultation was documented though the IT practice program. The pharmacist recorded his/her actions in the program. Evidence for those cases where the community pharmacists didn´t adhere to the guidelines, for example, when referral criteria was detected by the pharmacists but the patient was not appropriately refer and those cases where the pharmacists recommended modification of the treatment requested but the patient did not follow the recommendation were recorded as part of the process. |

1. Pharmaceutical Care Forum in Community Pharmacy. Practical Guide to Pharmaceutical Care Services in Community Pharmacy. Madrid: General Pharmaceutical Council of Spain; 2019. [↑](#endnote-ref-1)
2. Dineen-Griffin S, Benrimoj SI, Rogers K, Williams KA, Garcia-Cardenas V. Cluster randomised controlled trial evaluating the clinical and humanistic impact of a pharmacist-led minor ailment service. BMJ Quality and Safety. 2019: 1–11. Doi:10.1136/bmjqs-2019-010608. [↑](#endnote-ref-2)
3. Paudyal V, Watson MC, Sach T, Porteous T, Bond CM, Wright DJ, et al. Are pharmacy-based minor ailment schemes a substitute for other service providers? A systematic review. British Journal of General Practice. 2013;63:472-81. Doi: 10.3399/bjgp13X669194. [↑](#endnote-ref-3)
4. Watson MC, Ferguson J, Barton GR, Maskrey V, Blyth A, Paudyal V, et al. A cohort study of influences, health outcomes and costs of patients’ health-seeking behaviour for minor ailments from primary and emergency care settings. BMJ Open. 2014;5:e006261. Doi:10.1136/bmjopen-2014-006261. [↑](#endnote-ref-4)
