# Supplementary material for: A cost utility analysis alongside a cluster-randomised trial evaluating a minor ailment service compared to usual care in community pharmacy
Source: BMC Health Serv Res. 2021 Nov 20;21:1253. doi: 10.1186/s12913-021-07188-4 (PMC8605551; doi:10.1186/s12913-021-07188-4)
Supplement: Supplementary file 3 — Additional file 3. Baseline characteristics for the sample by pharmacy group. [file 12913_2021_7188_MOESM3_ESM.docx]

Baseline characteristics for the sample by pharmacy group

|  | | **MAS CP* (n=323)** | **UC CP* (n=485)** | **Total** | **p-value** |
| --- | --- | --- | --- | --- | --- |
| Gender | Male  Female | 125 (38.7%)  198 (61.3%) | 173 (35.7%)  312 (64.3%) | 298 (36.9%)  510 (63.1%) | 0.382 |
| Education^†^ | None/Primary/Not know  Secondary  Superior | 102 (46.2%)  73 (33.0%)  46 (20.8%) | 140 (49.3%)  84 (29.6%)  60 (21.1%) | 242 (47.9%)  157 (31.2%)  106 (20.9%) | 0.691 |
| Employment^†^ | Employed  Unemployed  Retired  Student  Other | 112 (49.3%)  29 (12.8%)  48 (21.1%)  9 (4.0%)  29 (12.8%) | 172 (59.3%)  21 (7.2%)  46 (15.9%)  14 (4.8%)  37 (12.8%) | 284 (54.9%)  50 (9.8%)  94 (18.2%)  23 (4.4%)  66 (12.7%) | 0.124 |
| Health insurance^†^ | Public  Private/Both/Not known | 200 (87.3%)  29 (12.7%) | 253 (87.5%)  36 (12.5%) | 453 (87.4%)  65 (12.6%) | 0.944 |
| Consultation type | Symptom presentation  Direct product request | 235 (72.8%)  88 (27.2%) | 329 (67.8%)  156 (32.2%) | 564 (69.8%)  244 (30.2%) | 0.136 |
| Minor ailment | Upper respiratory  Pain  Digestive  Dermatological | 220 (68.2 %)  65 (20.1%)  24 (7.4%)  14 (4.3%) | 309 (63.7%)  96 (19.8%)  52 (10.7%)  28 (5.8%) | 529 (65.5%)  161 (19.9%)  76 (9.4%)  42 (5.2%) | 0.309 |
| First time symptoms | Yes  No | 26 (8.0%)  297 (92.0%) | 42 (8.7%)  443 (91.3%) | 68 (8.4%)  740 (91.6%) | 0.417 |
| Symptom already treated | Yes  No | 61 (18.9%)  262 (81.1%) | 110 (22.7%)  375 (77.3%) | 171 (21.2%)  637 (78.8%) | 0.196 |
| Other health problem/s | Yes  No | 148 (45.8%)  175 (54.2%) | 222 (45.8%)  263 (54.2%) | 370 (45.8%)  438 (54.2%) | 0.989 |
|  | | **Average (SD)** | | | **p-value** |
| Age (years) | | 48.1 (15.8) | 47.3 (17.1) | 47.6 (16.6) | 0.552 |
| Baseline EQ-VAS | | 68.2 (19.0) | 71.3 (19.6) | 70.1 (19.4) | 0.005‡ |
| Baseline utility | | 0.87 (0.12) | 0.89 (0.14) | 0.88 (0.13) | <0.001‡ |
| Symptom duration (days) | | 3.6 (3.7) | 3.9 (4.4) | 3.8 (4.1) | 0.263 |
| Nº medicines to treat other health problems | | 1.2 (1.9) | 1.3 (1.9) | 1.2 (1.9) | 0.798‡ |

* MAS CP: Minor Ailment Service Community Pharmacies; UC CP: Usual Care Community Pharmacies; SD: Standard deviation

† Data recorded 10 days after consultation by phone, 291 patients answered in CP control group (60.0%) and 229 in CP intervention group (70.9%)

‡ Mann-Whitney
